# The Works of John Hunter, F.R.S.; with Notes

**Published:** 1837-07

**Authors:** 


### Art. V.

*The Works of* John Hunter, f.r.s.; *with Notes.* Edited by James F. Palmer, Senior Surgeon to the St. George's and St. James's Dispensary, &c. &c. *In Four Volumes, illustrated by a Volume of Plates, in quarto.—London,* 1835. Vol. I. 8vo. pp. 643.

In our last Number we gave a brief notice of the edition of Hunter's works, of which the first volume is now before us. This volume contains the Life of the author by Mr. Drewry Ottley, and the surgical lectures delivered by John Hunter in the year 1786-7, and now first published from ms. copies taken by some of his pupils at the time. In our next number we hope to notice these lectures: on the present occasion we shall confine our attention exclusively to the memoir of Mr. Ottley.

The time which has elapsed since the death of John Hunter, is favorable to the drawing up of an accurate and faithful memoir of him. Nothing seems more rare than a candid and impartial biography of any man who has recently died. It is sometimes undertaken to flatter or soothe the survivors, sometimes to commemorate the friendship of the biographer, and sometimes to gain reputation on the credit of the deceased. Of the biographers, some are mere panegyrists, and some display the spleen and vanity of rivals whose inferiority derives consolation from abating the merits of the departed. The great genius of John Hunter exposed his memory to most of these accidents: his tomb was covered with praises, some only of which were just; and assailed with detraction from which his undeniable greatness ought to have spared him. He received posthumous honours from a few who sought only to mortify others who had been the rivals of him, and of whom they were the rivals; and Mr. Jesse Foot took the trouble of writing a life of him, in order, apparently, to shew the deluded world how much it had erred in deeming him a great man.

There is nothing uncharitable in the supposition that Sir Everard Home had his own reasons for making the history of his departed patron uninteresting; for it is unquestionable that he meant to assume no small part of the true fame of John Hunter to himself. Dr. Adams, on the other hand, is accused of displaying everywhere marks of indiscriminate and unbounded partiality.

In all these cases, time is still "the avenger;" abating unmerited praise, withering the short-lived efforts of envy, and establishing just fame. Contemporaries may magnify or abuse, but posterity judges calmly; exempt, in the case at least of private individuals, from the delusions either of the good and evil passions, and therefore capable of estimating the life and the character at their real value.

John Hunter was the son of a small farmer at Long Calderwood, about eight miles from Glasgow, and the youngest of ten children. He was born in 1728. We smile at the assertion of his being "descended from the ancient family of Hunter of Hunterston," and derive yet more amusement from Mr. Wardrop's astonishment (expressed in the Life of Baillie,) at the extent of talent united in Dr. Baillie's (the nephew of Hunter) family and connexions, seeing that he makes it imparted by marriage to Sir Richard Croft, who married Mrs. Baillie's sister, and to Mr. Denman, who "was Dr. Baillie's brother-in-law." These commencements are like the invocations of the muse in the beginning of an Epic; equally trifling and equally fanciful; but indeed the blame of them rests, as we know from some experience of our own, less with the biographer than with the friends of the persons to be commemorated, who are dissatisfied to have merely to boast of a genius in the family, and always desiderate a fair genealogical tree. The names of William Hunter and of John reflect back on their humble ancestors far more honour than they could borrow from the Hunters of Hunterston, even if they were proved to have been hunters ever since the days of Nimrod.

We are almost again inclined to accuse Mr. Drewry Ottley of a desire to speak too magnificently when he mentions John Hunter, a boy of ten years of age, engaging, after the death of his father, " in country sports," and going, at the age of seventeen, with little preparation but such sports, " to the house of his brother-in-law Mr. Buchanan," (which Mr. Buchanan was a cabinet-maker in Glasgow), " under the hope of being able to assist in freeing him" (the said Mr. Buchanan,) " from the pecuniary difficulties into which his convivial habits and inattention to business had led him." We cannot affect surprise that this hopeful scheme, admitting it ever to have entered the mind of Buchanan or his nephew, was unavailing, and that " Mr. Buchanan soon after resigned his business," and became a teacher of music and clerk to an Episcopalian chapel. The object of this romantic story is, we suspect, to free the immortal John from the suspicion of having worked in the worthy cabinet-maker's shop; whereas, in truth, if John had worked at a cobler's stall his fame would not have been deprived of a single ray of brightness. " It is probable," says Mr. Drewry Ottley, with a sensitive regard for the Hunters of Hunterston, " that whilst here, Hunter, who prided himself on his manual dexterity, assisted his brother-in-law in his workshop." So it seems that John, after seven years spent in rural idleness, went to Glasgow to reclaim his uncle's affairs, settle his books, and give him his valuable *assistance* in the making of cabinets. Truly we think quite as well of the old version, which Mr. Ottley discards; and can well believe that at seventeen years of age, John, who had a rooted dislike to the Latin accidence and Corderius, and was muscular and active, and somewhat at a loss how to provide for himself food and raiment, repaired to his uncle to be instructed in his trade. So honest an intention needs no colour of excuses.

Meantime, William Hunter, his elder brother, who was fond of learning, was studying at Glasgow, was the pupil of Cullen, (then a practitioner at Hamilton,) and laying the foundations of his future distinction. In Mr. Ottley's account of William, the magniloquent is still too conspicuous. The " family property" had fallen to him, but " his mother continued, with his permission, to reside on the estate;" and yet William Hunter, good man, on receiving seventy guineas as entrance fees after the delivery of his introductory lecture, unaffectedly remarked that he had never been master of such a sum before. The virtuous poverty from which such a man sprung, his industry and perseverance, and their astonishing results, render his name far more illustrious than any descent from privileged freebooters, even if he had attained the dignity of a stupid country gentleman, rich in money and in lands, and a justice of the peace. It is strange that in English biographies these puerilities always meet us.

No nation is so trading, or so ashamed of trade. Foreigners often and justly remark, that with us, talent gives no place in society, confers no rank, unless associated with wealth, or supported by the supposition of high connexions.

The slumbering ambition of John Hunter was soon roused by the success of his brother William in establishing a school of anatomy in London. He scorned his "country sports," abandoned all ambition as to cabinet-making, forsook the auditing of distracted accounts, if such had ever filled or plagued his mind, and, being now twenty years of age, wrote to his brother and offered his services in the dissecting-room, little dreaming what triumphs there awaited him. Assisted in anatomy by William Hunter, and instructed in surgery by the great Cheselden, at the Chelsea hospital, his extraordinary powers were gradually called into exercise; the society of his brother's associates contributed to stimulate and to inform his mind; and his natural energy made him the delight of students and resurrection-men. His strong understanding was further improved, we may suppose, by his observation of the practice of Pott, at St. Bartholomew's; for, although that celebrated man was not yet so well known as he afterwards became, he appears to have been already among the first to simplify the practice of surgery, by a reference to the objects and efforts of nature.

In 1753, when John Hunter was twenty-five years of age, the notable design of polishing him into a scholar seems to have been entertained by his brother; and he "entered as a Gentleman Commoner at St. Mary's Hall, Oxford." But John's proud and rugged spirit was not to be tamed by the classics, and he never looked back upon this attempt with much gratitude. "They wanted to make an old woman of me," was his remark to Sir Anthony Carlisle many years afterward, "or that I should stuff Latin and Greek at the University, but these schemes I cracked like so many vermin as they came before me;" and John suited the action to the word. In fact, he had been the hero of the medical and of the dramatic theatre among the pupils, assisting them to damn dull plays in the gallery, and known familiarly among the dissectors as "Jack Hunter," and he was resolved that no one should make Jack a gentleman.

About this time he attached himself to St. George's Hospital, the only institution which held out to him a reasonable prospect of an appointment; and here he became well known as an anatomist, and pursued those investigations into the mode of connexion between the placenta and uterus, which ended in the discovery which twenty-five years afterwards so unfortunately became the subject of the rival claims of his brother and himself, in the assertion of which these distinguished men exhibited an animosity that has too often disgraced the minds of men of science. For three years these brothers maintained their mutual unforgiveness; John seems to have most regretted the estrangement, but William left the world unappeased.

Yet nature appeared, in her contrasted gifts to these two great anatomists, to have designed them to act through life in fraternal copartnery. William was learned, eloquent, and polished, and John was unrivalled in the dissecting-room. John was fitted to labour untired in dissection and physiological observation, and William to apply these labours to practice, or to embody their results in lectures calculated to spread the knowledge over all Europe; whilst, for a love of science, for philosophical habits of thought, and for mental power, they were equally remarkable. Thus prepared, notwithstanding the jealousy which each perhaps entertained of the other, they did in fact labour for many years and on many points in unison. John's indefatigable dissecting knife was continually laying open to him some of the mysteries of animal bodies; and when other anatomists, more learned than he, happened to arrive at the same point at the same time with him or his brother, the sharp and polished pen of William defended their claims with great power and astuteness. Hence arose the mighty feuds with the Monroes concerning the injection of the tubuli testis with mercury, and the true office of the lymphatics; hence the war with the amiable and accomplished Pott, whom they accused of stealing from them the knowledge of the true nature of congenital hernia; the truth being that Haller had in this particular preceded them all three. Such are the fretful labours of controversialists, who allow the love of fame to predominate over the pure love of truth: but here, also, posterity does justice to all parties. No one now wishes to disturb his mind by reading the disputes which engaged the pens and embittered the lives of so many of the physiologists of the last century, and yet there is perhaps no one of them to whom the present age is unjust. It would be well if some of the living would profit by this lesson; and, calmly pursuing truth for its own sake, would leave their merits to the decision of the discerning, confident of the result. Their restless obtrusion of their real or supposed discoveries, their fidgety fear of being run over and forgotten, their challenges and reclamations, their attacks and replies and rejoinders, whilst they doubtless disqualify them for being sound sleepers, neither amuse, nor instruct, nor convince, but make the paths of science thorny and unpleasant, which should be full of peace and delight.

Far more agreeable is it to turn from the contemplation of these quarrels to the quiet pursuits of John Hunter, to his close consideration of the descent of the testis in the fœtus, first by him accurately described; and to the commencement of his observations, and those perhaps of many others, on the hidden functions of the nervous system, by the patient tracing of the ramifications of the first pair of nerves within the nose; and to his ingenious and valuable experiments to determine the disputed question of venous absorption. Thus engaged, and after ten years of application, he began to turn his attention to the often-needed illustration to be derived from comparative anatomy; but the proud and extensive products of this direction of his mind were yet for a time withheld from him and from science by an attack of inflammation of the lungs (in 1759), "which left behind it symptoms which threatened to end in consumption." Being advised to seek a warmer climate, he obtained the appointment of staff-surgeon in the army, and accompanying the armament, under General Hodgson and Commodore Keppel, to lay siege to Belleisle, in 1761, "was at once furnished with ample opportunities for practice in the treatment of gun-shot wounds." In the following year he was with the army in Spain; and he did not return to London until 1763. During this part of his life, as at all other times, he was continually observing and reflecting. He instituted experiments on lizards and snakes, "to ascertain whether digestion continues during their torpid state;" and entered on some enquiries concerning the faculty of hearing in fishes. In whatever circumstances he was placed, he was still the interrogator of nature, and she answered him, as she answers all who question her with humility and zeal, eloquently well.

Returning to London, and now five and thirty years of age, John Hunter had to begin the world. The situation he formerly held with his brother had been filled up by Hewson, and his only fortune was his half-pay. He lived privately and economically, waited long for practice, and devoted much of his time, and much of his slender pecuniary means, to the pursuit of anatomical and physiological science. His manners were not such as powerfully to recommend him, and it was many years before he wrought his arduous way to a place beside the most illustrious surgeons of the time. He seems to have been ignorant of, or to have despised those "minor tactics," as his biographer terms them, which often concur largely to what is called getting on in the world.

"But after all, perhaps, the principal reason why Hunter was so long in obtaining a large share of practice was, that he looked not, as most men do, to the acquisition of fortune as the end for which he was labouring; but, on the contrary, considered wealth only as a means by which he might advance the far more important objects he had in view. His powerful mind was unceasingly stimulated by an ardent desire to forward the acquisition of those branches of knowledge which to him appeared best fitted to promote the improvement of his profession; to this object was devoted every hour that he could spare from his daily avocations, or snatch from the time allotted by others to sleep; and to promote this end he was always ready to sacrifice the claims of worldly prudence and self-interest. To witness an interesting or extraordinary case he would take any trouble, or go almost any distance, without a chance of pecuniary recompense; but to the daily routine of practice he always returned unwillingly, and even when he had acquired a lucrative and extensive business, he valued it only as affording him the means of pursuing his favorite studies. This feeling he would often express to his friend Mr. Lynn, when called to see a patient, by saying, as he unwillingly laid by his dissecting instrument, "Well, Lynn, I must go and earn this d—d guinea, or I shall be sure to want it tomorrow." (P. 27.)

It may perhaps console some living lecturers to know, that, at this time, John Hunter's lectures on anatomy and operative surgery were delivered to an audience never exceeding twenty. But for this kind of disappointment, he enjoyed abundant consolation in the continual delight he experienced from the prosecution of comparative anatomy. The tigers in the tower were more interesting to him than the fees of pupils. Whenever he was master of ten guineas, he added to his museum. He allowed the proprietors of menageries a life-interest in rare animals, and was delighted to receive and examine the carcases when the animals died. In this devotion of himself to one great object, regardless of ordinary obstacles, we recognize the characteristic of a truly great man; and it adds to his merit, that even in the widest range of his enquiries he had ever a regard to illustrating the phenomena of disease as well as of health, thus making philosophy the handmaid of medicine and surgery.

"He clearly saw, that in order to obtain just conceptions of the nature of those aberrations from healthy actions which constitute disease, it was necessary first to understand well the healthy actions themselves; and these required to be studied, not in man alone, but throughout the whole animal series, and even to receive further elucidation by comparison with the functions of vegetable life. It was no less an undertaking, then, than the study of the phenomena of life, in health and disease, throughout the whole range of organized beings, in which Hunter proposed to engage; an undertaking which required a genius like his to plan, and from the difficulties of executing which, any mind less energetic, less industrious, and less devoted to science than his own, would have shrunk.

"In pursuing these researches, he strove not, like many of his more learned but less philosophic predecessors, to unravel the mysteries of nature by taking up some principle *a priori*, and seeking for facts to support his theory. On the contrary, he followed, in the strictest manner, the inductive method laid down by the great father of modern philosophy, as the only sure though arduous road to knowledge. He aimed not at discovering the essence of life, satisfied that this was beyond the province of philosophical research; but he sought to know how the various organs are constructed, and how they act in accomplishing those various processes by which the presence of this principle is manifested. Nor was he content to acquire his information at second hand. Instead, therefore, of referring to the discoveries detailed in books, he appealed directly to nature herself, and rested nothing upon the facts related by others, until, by the evidence of his own senses, he had ascertained their truth." (P. 31.)

In 1768, Hunter was elected surgeon to St. George's Hospital, and was enabled by this circumstance to take private pupils, among whom were the celebrated Jenner, the late deservedly esteemed Mr. Guy, of Chichester, Dr. Physick, of Philadelphia; and lastly, and as the sequel proved, least worthy, Sir Everard Home. In 1771, he published the first part of his Treatise on the Teeth. In the same year, he married the sister of Sir Everard Home, and, although his wife's gay parties sometimes mightily discomposed him, the marriage seems to have been on the whole a very happy one. In 1772, he presented to the Royal Society, of which he had been already five years a member, a memoir on the digestion of the stomach after death, by the gastric fluid contained in it; a fact which he was the first to point out, and which the researches and experiments of Dr. Carswell have fully established.

He had now filled all the principal rooms of his house with preparations. Three or four hours before breakfast, and as much time during each day as could be spared from practice, he devoted to his museum. His practice increased also, but yet before 1774, when he was forty-six years of age, it did not amount to a thousand a year. A singular account is given of an attack of illness which he had about this time, consisting of spasm, in the region of the pylorus, attended with a cessation of the heart's action for three-quarters of an hour. We believe, although the memoir before us does not say so, that his attention was first drawn to his pulse by remarking the extraordinary paleness of his face in the glass. His sensations and voluntary actions were during this time unimpaired, and "he continued to respire by a voluntary effort, with a view of keeping himself alive." It is presumable that the cessation of the heart's action was not complete, and that careful auscultation would have detected feeble contractions.

In this year (1775), Hunter first delivered a course of lectures exclusively on surgery. His dislike to delivering public discourses was extreme; and whenever he gave an introductory lecture, he previously took laudanum. Although he was never a popular lecturer, and had no pretensions to elegance of diction, there must have been something striking in the originality and force of his observations. Yet it sounds oddly to be told, as he was in the habit of expressing himself, that a ball had "gone into a man's belly and hit his guts such a d—d thump, that they mortified."

We shall not attempt to enumerate Hunter's writings, or his contributions to the Transactions of the Royal Society, which were numerous and valuable. The Chronological list contains fifty-five separate memoirs, in various publications. On every subject his ardour of enquiry is conspicuous, and it is the ardour not of an anatomist and physiologist merely, but of a naturalist in the widest sense of the term. Although greatly inferior to Cuvier in accomplishments, he was, like that great man, distinguished by the most laborious application of time to similar subjects; and, if at that time any public encouragement had been given to such pursuits, he would doubtless have founded a magnificent national establishment of Natural History in England. For this, however, he prepared the way; although only by the devotion of his life to the subject, and not a less sum altogether than 70,000*l.*; an enormous and memorable sacrifice for a man whose only estate was his industry.

He was generally in his dissecting-room before six in the morning, and his evening studies were often prolonged an hour or two after midnight. His days were necessarily devoted to seeing patients, in the course of which duty his punctuality was exemplary.

—— "he kept a regular entry of his engagements in a book at home, and carried an exact copy of this in his waistcoat pocket, so that by a reference to the book he could always be found at any hour of the day, in the event of his being wanted. Any unnecessary discomposure of these engagements greatly annoyed him, and caused him to give vent to his feelings in no measured terms. The late Mr. Cline once excited his ire by an offence of this kind. He had engaged Hunter to meet him in consultation on a case in the afternoon, but in the course of his morning rounds saw another patient, respecting whom he wished to take Hunter's opinion, and accordingly, without giving him previous notice, appointed to call with him after the former engagement was ended. When the first visit was over, Cline mentioned the second appointment, on learning of which Hunter got into a towering passion, and asserted that Cline had acted in the most unjustifiable manner in thus deranging the whole of his arrangements for the afternoon. Cline, who was of a very placid temper, was amazed to see such a storm excited by so trifling a cause, and said what he could to appease it. In this he succeeded, and Hunter, soon recovering himself, turned to him, and in a very altered tone said, "Come along, then, let us go and see our patient." Hunter was equally strict in enforcing punctuality on his household: he dined at four, then the fashionable hour, and gave strict orders that dinner should be ready punctually whether he was at home or not. He was a very moderate eater, and set little value on the indulgences of the palate. During many of the latter years of his life he drank no wine, and therefore seldom remained long at table after dinner, except when he had company; but then, though he abstained himself, he was not willing to allow his friends to follow his example.

"After dinner he was accustomed to sleep for about an hour, and his evenings were spent either in preparing or delivering lectures, in dictating to an amanuensis the records of particular cases, of which he kept a regular entry, or in a similar manner committing to paper the substance of any work on which he chanced to be engaged." * * * "At twelve the family went to bed, and the butler, before retiring to rest, used to bring in a fresh argand lamp, by the light of which Hunter continued his labours until one or two in the morning, or even later in winter." (P. 52.)

With such habits, it becomes conceivable that he should have found time, amidst the distractions of practice, to form his noble museum, which we trust is at length likely to be made useful to the public. His letters to Jenner are curious for their brevity, for the anxiety they evince on every subject connected with natural history, and for the confidence they shew him to have felt in his own powers of exertion, and in those of other men. "I thank you," he says in one of them, "for your experiment on the hedgehog; but why do you ask me a question by way of solving it? I think your solution is just; but why think? why not try the experiment? Repeat all the experiments on a hedgehog as soon as you receive this, and they will give you the solution. Try the heat: cut off a leg at the same place: cut off the head, and expose the heart; and let me know the result of the whole. Ever yours, J. Hunter." Indeed, in some of his letters he gives Jenner at least a month's task, and though not literary, he always employs a style of his own, and which plainly derives its character from that of his thoughts. Sometimes he mixes his topics oddly enough. "I have but one order to send you," he says on another occasion, "which is to send every thing you can get, either animal, vegetable, or mineral, and the compound of the two, either animal or vegetable, mineralized. I would have you do nothing with the boy but dress him superficially: these funguses" (cerebral) "will die, and be damned to them, and drop off. Have you large trees, of different kinds, that you can make free with? Have you any eaves, where bats go at night?" &c.

When engaged in some experiments on the heat of animals and vegetables, "with a view to discover if it were possible to restore to life animals which had been frozen," he became enamoured of his subject, and speculated "on the possibility of freezing human beings, and thawing them to life two or three centuries after, a project which," Mr. Ottley says, "if he could realize, he expected would make his fortune." We may imagine with what interest he would have received the astounding fact, lately announced by Mr. Crosse, that insects fossilized in flint may be revivified after a thousand years of stony torpor.

Examples of almost superhuman industry, like that exhibited by Hunter, are often held up for imitation, too little qualified by the reflection that as it surpasses the endurance of the majority of men, any attempt to imitate it is commonly followed by grievous penalties. Even Hunter's strong frame seems to have been early and seriously impaired; and, at least in some degree, in consequence of the perpetual employment and frequent excitement of his mind and feelings. To one severe attack allusion has already been made: another occurred in 1777, when, in consequence of mental disturbance, he was affected with vertigo for many days, attended with morbid acuteness of the organs of sense, and various nervous sensations. His friend Jenner was shocked by the appearance he presented after this attack, and suspected him to labour under angina pectoris, arising from some disease of the heart. In 1785, (we conjecture, for the biography is not aided by marginal dates), he suffered from a painful affection of the heart and arteries, and from spasmodic affections of the face, arms, chest and stomach, and finally "a violent spasm of the heart, which, after half an hour of agonizing pain, ended in syncope." At this time, however, he laboured under some apprehension of hydrophobia. These attacks were unfortunately induced by mental irritation, to which he was extremely prone. By the end of the year he had recovered sufficiently to plan and carry into execution "his famous operation for the cure of aneurism; that of tying the artery at a distance from the tumour, and between it and the heart, instead of laying open and emptying the sac, and then seeking for the orifices of the vessel, according to the old operation." His biographer endeavours, with much candour and fairness, to determine the precise amount of credit due to Anel and Desault in connexion with this operation.

As we propose giving a distinct account of the principal works of John Hunter, we purposely pass over several notices of his publications interspersed with the biographical memoir, dwelling chiefly on the particulars of his life, which, like that of most men of science and great application, was not very eventful. We find him ever pursuing the same industrious course, ever displaying the same desire to become the possessor, almost at any price, of whatever could illustrate the animal economy, or, indeed, any of the kingdoms of nature. For this purpose he toiled, for this he built, for this he expended all the money he could command; in short, for this he lived. He let none of his friends rest idle or unsolicited who were capable of helping him or adding to his stores; he sent out agents to the north seas to procure information concerning whales; and he made it the business of one of his men to watch the declining health of the Irish giant O'Brien, that his bones might adorn his museum. Poor O'Brien did not participate in this zeal for the illustration of osteology, and died assured that he should be enclosed in a leaden coffin and sunk in the sea. Hunter's man, however, bribed the watchers of the corpse with drink, until a bargain was made to surrender up the body to Hunter, at the enormous price of five hundred pounds.

Those who possess, or have seen and admired Sharp's engraving of John Hunter, will be interested to learn that John seems to have submitted to the great privilege of being painted by Sir Joshua Reynolds with almost as bad a grace as he would have consented to construct bad Latin verses at Oxford. He proved a troublesome sitter; but at length, probably tired of the operation, fell into a train of thought in the attitude in which the portrait represents him, an attitude so preferable, it would appear, to that in which he was originally intended to be exhibited, that Sir Joshua turned his half painted canvass upside down, and made a fresh sketch, of which the head was between the legs of the former.

When the biographer of Hunter has advanced as far as the sixtieth year of this indefatigable man, we are not surprised that he is enabled to represent him as one of the first anatomists, physiologists, and surgeons of the day. Every page of the life still contains testimony of his unabated zeal for science, and of his encouragement of it in younger men. He was continually enriching his profession with the fruits of his research and meditation, and in the midst of innumerable engagements of the study and of active life, was carefully preparing the great work on Inflammation and Gun-shot wounds; which, notwithstanding occasional obscurities of style, will ever rank among the classical productions of medicine, and meditating a still larger work on his museum. His character had naturally become at this period of his life more broadly impressed with peculiarities, which were, however, "of a mingled yarn." His generosity as to money matters was remarkable. His sentiments with respect to the concealment of the nature of successful medicines was a less pleasing trait. Although continually engaged in the contemplation of the works of the Creator, his mind never seems to have been raised beyond the visible phenomena of life, and his biographer would have done wisely to omit his scoffing assent to become godfather to one of Jenner's children. To the calmness of a philosopher he had no pretensions; yet he bore detraction patiently. The tender emotions were unknown to him, but he felt for his friends in their misfortunes, and was prompt to relieve them. It is as a man of science that Hunter is entitled to admiration, and in this respect his claims are of the highest kind. Of all the privileges of superior minds none is so noble, none so truly worthy of desire, as that of influencing the minds of others for many generations; and this influence we believe will be attached to the name of Hunter. There are few subjects in physiology, especially, which did not at some time or other employ his thoughts; and wherever in his writings he fails to elucidate, his strong understanding has yet thrown a ray by which his followers may be lighted to further discovery.

We live in days when science is in favour, but it requires no great effort of imagination to conceive how Hunter's habitual employments would be regarded and represented, by the mere *practical* men of his day, (as those of the most moderate acquisitions generally term themselves,) as inconsistent with the qualifications required in a good surgeon. In a knowledge of the principles of surgery, it will be conceded that few were his equals; and if he did not excel in the performance of operations he never failed, aided by his sound anatomical science, to bring any operation which he undertook to a perfect conclusion. But there are many narrow-minded men in all professions, who regard books and study with a kind of detestation, and who console themselves for conscious deficiencies by boasting of their practical cleverness. In the mere manual parts of surgery they may possess something of this kind; but in its higher departments, and in the practice of medicine, such men are as ignorant at the bed side as they are in the study. Their best friend is the constant uncertainty of physic, which takes away confidence almost in proportion to men's powers of reflection, and leaves the illiterate prescriber in a world of imaginary triumphs. With some minds of this stamp John Hunter seems to have been associated at St. George's Hospital; men who hated him for his attainments, considered that surgery could be no further advanced, and proclaimed him an enthusiast and an innovator. One of them "did not choose to hazard his reputation in giving lectures;" another "did not see where the art could be improved;" and all united in opposing his plans for making the hospital more useful as a place of education. The governors of the hospital sided with the ignorant majority, and left Hunter's suggestions to be adopted by another and a wiser generation. In contests of this kind, the advocates of illiberal measures have generally for a long time the advantage, for they defend their course by narrow views, which the meanest minds readily comprehend and sympathise with. The following passage details the principal manœuvre employed against Hunter, and its tragic result.

"A Committee was subsequently appointed to draw up a code of rules for regulating the admission and instruction of pupils; and a set of proposals was submitted to them by Mr. Hunter's colleagues, which was agreed to without his having been even consulted on the occasion! Many of the regulations adopted continue in force to the present day; others, and especially those relating to the better instruction of the pupils, soon fell into disuse; and some seem to have been especially directed against Mr. Hunter. Amongst these latter, was one which determined that for the future, no person should be admitted as a student of the hospital without bringing certificates that he had been educated to the profession; a regulation which was probably designed to exclude Mr. Hunter's countrymen, who sometimes came up to town recommended to him, and entered as his pupils at the hospital, without having had any previous medical instruction. Nor was this clause long in taking effect; for in the autumn two young men, who had come up to town ignorant of this new regulation, applied to Hunter to be admitted under him at the hospital. He informed them of the law which had been passed, but undertook to press for their admission at the next Board-day, and directed them to furnish him with a statement of their case in writing. On the 16th of October the Board was to meet, and Hunter prepared to fulfil his promise though he was so well aware of the risk he incurred in undertaking a task which he felt would agitate him, that in mentioning the circumstance to a friend who called on him in the morning, he expressed his apprehension lest some unpleasant dispute might occur, and his conviction that if it did it would certainly prove fatal to him. At his accustomed hour he left his house to commence his morning rounds, and by accident forgot to take with him his list of appointments; he had left the house but a few moments when it was discovered, and Mr. Clift, who was then residing in his house, hastened with it to York Street, St. James's, the first place on the list, where he found the carriage waiting. Hunter soon made his appearance, took the list, and in an animated tone called to the coachman to drive to St. George's. Arrived at the hospital, he found the Board already assembled, and entering the room, presented the memorial of the young men, and proceeded to urge the propriety of their being admitted. In the course of his remarks, he made some observation which one of his colleagues thought it necessary instantly and flatly to contradict. Hunter immediately ceased speaking, retired from the table, and struggling to suppress the tumult of his passion, hurried into the adjoining room, which he had scarcely reached when, with a deep groan, he fell lifeless into the arms of Dr. Robertson, one of the physicians of the hospital, who chanced to be present. Dr. Baillie had immediately followed him from the Board-room, and Mr. Home, who was in the house, was also summoned to his assistance. Various attempts were made for upwards of an hour to restore animation, under the hope that the attack might prove to be a fainting fit, such as he had before experienced, but in vain; life had fled; and all their efforts proving useless, his body was placed in a sedan chair and conveyed to Leicester-square, followed by his now vacant carriage." (P. 130.)

Thus suddenly closed the life of this remarkable man, in his sixty-fifth year. Recollecting the circumstances of Mr. Hunter's previous health, the appearances found in the heart after death possess great interest. The pericardium was much thickened: the heart was small, the result of wasting and not of contraction of its fibres; two opaque white spots were found on the left auricle and ventricle: the muscular structure of the heart was pale and loose in its texture: the coronary arteries were with difficulty divisible by the knife. The mitral valves were ossified. There was some dilatation of the aorta, its valves were somewhat rigid, and the inner surface of the artery was studded with opaque and elevated white spots.

In the summing up of Mr. Hunter's character, Mr. Ottley exhibits equal judgment and candour; we do not, however, think it necessary to quote more from this very interesting memoir, which, with the valuable works now first collected, and carefully edited, with the addition of useful notes, will we trust find its way into every public medical library, and into many private collections of medical literature.

It is painful to look back on the difficulties which both the Hunters experienced, in rousing the feelings of the English public to any demonstration of zeal for science. William Hunter offered at one time to lay out 7000*l.* in the foundation of a national museum, but could get no countenance from government; and John's unrivalled collection, after his death, and the usual miserable process of bargaining, was purchased for less than half its value, and placed where it was long of little advantage to the profession or the public. The warlike ministry of that season of national insanity, were astonished at a request to encourage science. "What!" exclaimed Mr. Pitt, buy preparations! why, I have not money enough to purchase gunpowder." No encouragement would be afforded to the sciences which preserve life, by those whose thoughts were chiefly turned to the most compendious methods of killing men. We have no reason to be proud when we recollect that the French government, though equally steeped in human blood, was at that time fostering science with the utmost care, and rewarding its professors with unexampled liberality.

The great monument of John Hunter is the Hunterian Museum; far more honorable to him than "storied urn or animated bust." Part of its utility must long, we fear, be withheld by the want of those references which were burnt by Sir Everard Home. It is needless, now, to add to the execrations which are uttered over Sir Everard's memory. Meanness, ingratitude, and dishonesty could go no further than he carried them. But we think he is yet too highly estimated as a man of science; and we know that in conversation at meetings professedly scientific, his sentiments and language were so gross, as to justify a belief that his attainments were as limited as his mind was impure. We allude to this circumstance, not to add to the deserved obloquy which covers his character, but to warn students of youthful age against the worship of such unclean deities, whose temporary influence is often great, but never salutary. But Sir Everard Home could only destroy the manuscripts of the master to whom he was so deeply indebted. The preparations and specimens yet remain, and for the most part speak for themselves. Hunter's truly great design is stated to have been to illustrate all the phenomena of life in all organized beings. As he proceeded, he became interested in the fossil remains of forms of life no longer existing. The illustration of diseased actions was another great object; and this was effected by every aid that industry or art could employ. In time the collection grew so immense that he despaired of living to finish its arrangement, and to write a fair exposition of it. Never was there, therefore, presented to a surviving friend and pupil so grateful and so noble a task as that of completing a work, to leave which unfinished was one of Hunter's chief regrets when he found his health declining. Like all other men of comprehensive minds, he found the great scene of nature continually opening before him, enlarging, and exhaustless; but human life is short, and the longest preparation is generally made for no more effective end than to leave the greatest designs incomplete. A second period of sixty years would have been wanting to Hunter, as to Cuvier, to arrange, to survey, to reflect upon and interpret, the materials gathered together in the space of life allotted to one man.

Other labourers remain, and the collection, improved, and exhibited to advantage, and explained by able followers, is yet open to the student; destined, we trust, to stimulate many minds to fresh acquisitions. The medical student, especially, cannot be too strongly urged to visit and revisit the museum. It should be seen again and again: whole books should be read in it, with its preparations for illustration. Days, weeks, and months should be spent in it. The Council of the College of Surgeons will almost compensate us for thirty years of curatorial sleep, if they will carry into full effect their supposed design of publishing descriptive catalogues of every part of the collection.

---

